# Regional and Temporal Differences in the Functionality of Facultative Vertebrate Scavenger Communities

**DOI:** 10.1002/ece3.73625

**Published:** 2026-06-03

**Authors:** Indy van der Giessen, Julia van Knippenberg, Marijke van Kuijk, Elke Wenting

**Affiliations:** ^1^ Department of Biology Utrecht University Utrecht the Netherlands; ^2^ ARK Rewilding Nederland Nijmegen the Netherlands; ^3^ Biodiversity Research Institute (CSIC—University of Oviedo—Principality of Asturias) Mieres Spain; ^4^ Radboud Institute for Biological and Environmental Sciences, Department of Ecology, Radboud University Nijmegen the Netherlands

**Keywords:** carrion ecology, functional diversity, scavenger diversity, scavenging

## Abstract

Carrion represents a nutrient‐rich but ephemeral resource that plays a disproportionate role in ecosystem functioning by supporting scavenger communities and nutrient cycling. However, we lack understanding of how facultative vertebrate scavenger communities are structured across space and time. We examined the functionality of facultative vertebrate scavenger guilds on a regional and temporal scale using a dataset collected in protected areas in three geographical regions in the Netherlands (Veluwe, De Hamert Estate and KempenBroek). We assessed scavengers' functional roles—defined as species‐specific contributions to carcass consumption—scavenger diversity, and carcass depletion times based on behavioural traits, feeding preferences, and carrion exploitation. Functional roles of facultative vertebrate scavengers were represented across the regions, although the occurrence of specific species was region‐specific. In the Veluwe, scavenging behaviour of red fox ( *Vulpes vulpes* ) varied between consecutive years, likely driven by differences in resource availability. Across the regions, higher scavenger diversity (Shannon index) was associated with longer carrion depletion time, reflecting that slower carrion decomposition allows more facultative scavenger species to detect and utilise the carrion. Our results demonstrate that the functionality of scavenger communities is shaped by both regional and temporal factors. This highlights the importance of continuously or periodically (re)evaluating the functionality of facultative vertebrate scavenger communities across regional and temporal scales.

## Graphical abstract

We examined the functionality of vertebrate scavenger guilds on a regional and temporal scale using a dataset collected in protected areas in three regions in the Netherlands, characterised by the absence of large carnivores and obligate scavengers. Our results demonstrate that the functionality of scavenger communities is shaped by both regional and temporal factors. This emphasises the importance of continuously or periodically (re)evaluating the functionality of regional scavenger communities across regional and temporal scales, given potential upcoming changes in scavenger communities due to carnivore re‐establishment.

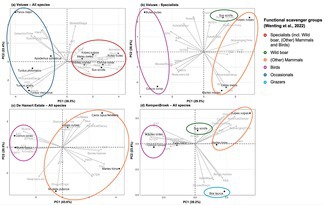

## 1 Introduction

Carrion—i.e., dead animal matter—is considered a nutrient‐rich and ephemeral organic resource, the decomposition of which forms a specialised and ecologically important process in ecosystems (e.g., DeVault et al. 2004; Carter et al. 2007; Wilson and Wolkovich 2011). Although carrion contributes less than 5% of the total mass of detrital input in terrestrial ecosystems, its role in shaping ecological communities is disproportionate compared to plant litter (Barton et al. 2013), by functioning as a nutrient hotspot (e.g., Keenan et al. 2018; Wenting et al. 2023; DeBruyn et al. 2025), and supporting diverse scavenger and decomposer communities (e.g., Barton and Evans 2017; Mateo‐Tomás et al. 2017; Bartel et al. 2024).

Scavenging—i.e., the consumption of dead animal matter—is a widespread feeding strategy utilised by a broad range of species (Wilson and Wolkovich 2011). This includes vertebrate and invertebrate scavengers, that can be classified as obligate or facultative scavengers (Wilson and Wolkovich 2011). Obligate scavengers feed exclusively on carrion (i.e., vultures), while facultative scavengers consume carrion opportunistically alongside other food sources, such as red fox ( *Vulpes vulpes* ) and corvids (e.g., Wenting et al. 2022; Newsome et al. 2024; Marie Montenegro et al. 2025). Vertebrate scavengers play a particularly significant role in ecosystem function due to their ability to remove large quantities of carrion in relatively short time frames (Inagaki et al. 2025), thereby preventing or reducing the leaching of nutrients (Wenting, Jansen, et al. 2024). Here, we focus on facultative vertebrate scavengers.

Understanding how scavenger communities are composed can reveal information about their functioning in terms of efficient carcass removal (Sebastián‐González et al. 2021; Grootaers et al. 2025; Monar‐Barragán et al. 2025). Scavenger species can fulfil different functional roles within the community by feeding on different body parts and being active in different stages of decomposition (Wenting et al. 2022; Marie Montenegro et al. 2025). Consequently, different species may have different effects on the decomposition of a carcass, potentially resulting in different effects on, among other, the spreading of nutrients across landscapes (Wenting et al. 2022; Cabrera‐García et al. 2025).

These functional roles are reflected in species‐specific behaviours, time of scavenging, and preferences for particular animal tissues (Sebastián‐González et al. 2021; Inagaki et al. 2025). For instance, Young et al. (2014) demonstrated that, in the United Kingdom, common buzzards ( *Buteo buteo* ) were only present during the early stages of decomposition and primarily consumed soft tissues, whereas carrion crows ( *Corvus corone* ) were observed throughout the entire decomposition process, showing increased feeding activity in later stages and exploiting a wider range of carcass parts including fur. In the Netherlands, Wenting et al. (2022) determined functional groups based on scavenger behaviour (e.g., eating or passing), the timing of their visit, and when observed eating, their eating preferences. Conversely, it was found that common buzzards were mostly scavenging during a later decomposition stage, and classified carrion crows as one of the most prevalent scavengers that primarily exploited carrion during earlier decomposition stages. This disparity between studies of the same species indicates that scavenger behaviour may be highly dependent on local circumstances, which raises questions about how the functionality of local scavenger communities varies with regional and temporal scales.

In this study, we examined the functionality of facultative scavenger guilds at a regional, i.e., different geographic zones in the Netherlands, and temporal scale, i.e., in subsequent years. We used a dataset collected in Dutch protected areas (Wenting et al. 2022; Wenting, Jansen, et al. 2024), characterised by the absence of large carnivores and obligate scavengers, i.e., vultures, at the time of data collection. With large carnivores such as wolves recolonising more of their historical home ranges (Planillo et al. 2024) and vultures occurring further north more frequently (e.g., Safford et al. 2019), we considered this dataset appropriate and relevant for the purpose of our study. The Dutch scavenger community has be divided into functional groups, consisting of species that have been recorded to feed on carcasses to a greater of lesser extent, including: (i) Birds such as common buzzard, common raven ( *Corvus corax* ) and carrion crow; (ii) Wild boar ( *Sus scrofa* ); (iii) (other) Mammals, including red fox, beech marten ( *Martes foina* ) and European polecat ( *Mustela putorius* ); and (iv) Occasionals, including great tit ( *Parus major* ) and roe deer ( *Capreolus capreolus* ) (Wenting et al. 2022). In particular, Wild boar has been considered a separate functional group due to its high body mass, social nature and capability to speed up the decomposition speed once it starts scavenging (Wenting et al. 2022; Wenting, Jansen, et al. 2024). Furthermore, longer decomposition times, i.e., longer time windows of field exposure, have been associated with higher scavenger diversity (Wenting et al. 2022). Here, we focused on scavengers' functional roles and carcass depletion times related to scavenger diversity.

## 2 Methods

### 2.1 Study System and Scavenger Community

**FIGURE 1:**
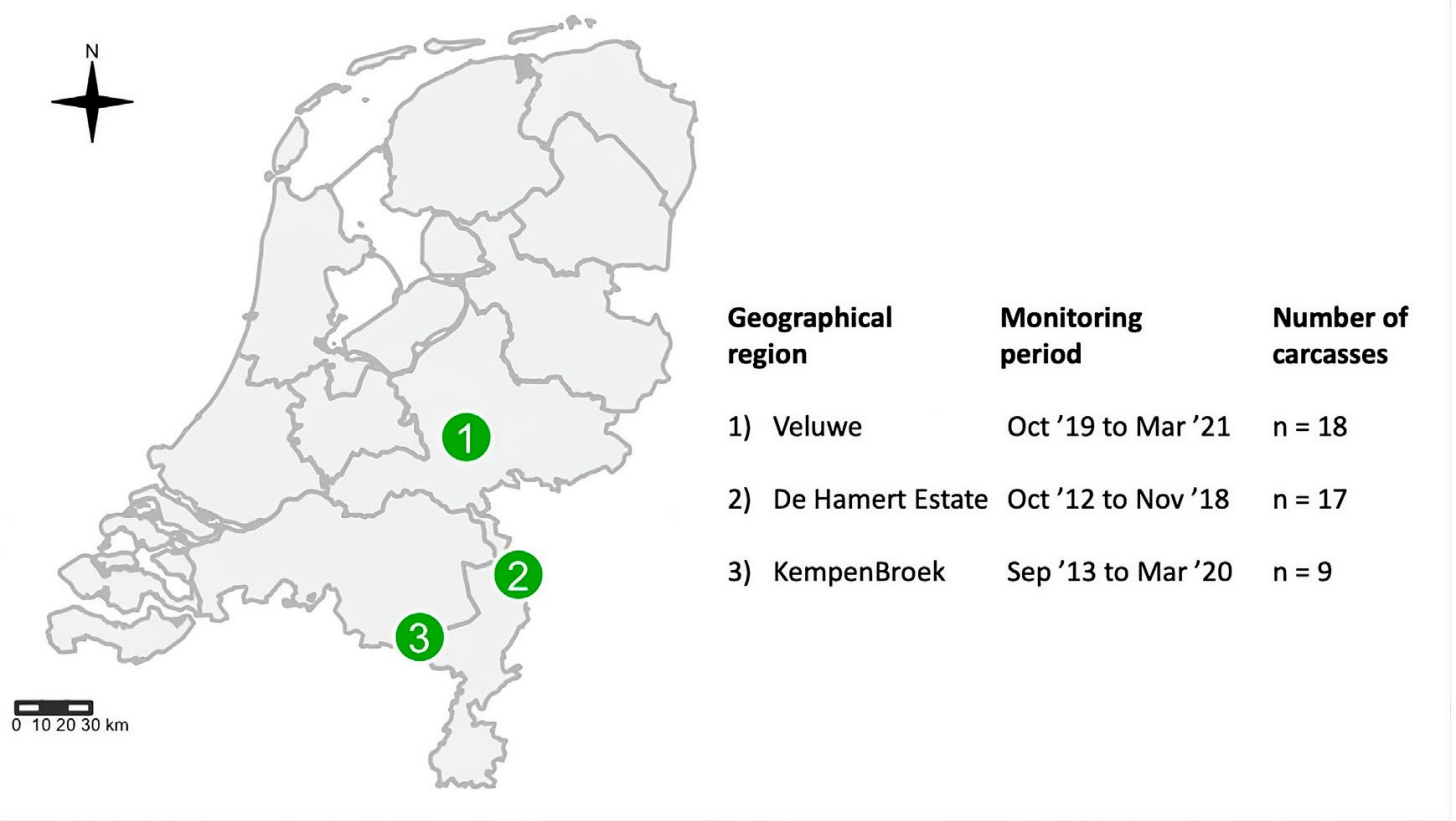
The selected regions we used to investigate the functionality of Dutch scavenger communities on a regional scale, including the monitoring period and the number of carcasses monitored.

We used data of vertebrate scavenger activity at 44 carcasses in three geographical regions—Veluwe, De Hamert Estate and KempenBroek—in the Netherlands. The data were collected between October 2012 and March 2021 (Figure 1 and Table S1; Wenting et al. 2022; Wenting, Jansen, et al. 2024). The exact monitoring period varied in each region (Figure 1). The carcasses were placed in heathlands or forested areas.

The Veluwe (52°10′ N 5°51′ E) is situated on glacier deposits and cover sands, characterised by sandy and loess soils. Ungulates such as wild boar, red deer ( *Cervus elaphus* ) and fallow deer ( *Dama dama* ) are abundant. A broad range of facultative scavengers are present, including wild boar, European pine marten ( *Martes martes* ), European badger ( *Meles meles* ), common raven, and common buzzard (Wenting et al. 2022).

The Hamert Estate (51°31′ N 6°10′ E) is a small‐scale river‐dune and peat landscape, formed by wind‐blown sands and deposits of the Maas river. Roe deer are the most common ungulate species. The region inhabits scavenger species such as red fox, common raven, and common buzzard (Wenting et al. 2022).

The KempenBroek (51°13′ N 5°39′ E) is a lowland wetland system, predominantly shaped by peat formation. It is home to ungulates such as roe deer and free‐roaming cattle. Facultative scavenger species include wild boar, beech marten, and common buzzard (Wenting et al. 2022).

### 2.2 Data Collection

We used motion‐triggered infrared camera traps deployed at fresh carcasses (for all details, see Wenting et al. 2022; Wenting, Jansen, et al. 2024). All camera traps were part of the Bushnell Trophy Cam product line. The camera traps were attached to trees or actively placed on poles at two meters from the carcass at one‐meter height and pointing toward the ground. The camera traps recorded videos of 60 s per trigger with a two‐ or three‐second interval between triggers depending on the model.

Carcasses were positioned with the abdomen or back to the camera and tied by the legs to trees or poles to prevent the carcasses from getting dragged out of view. All the carcasses in this study were freshly obtained from regular culling or roadkill, that is, no animals were killed for the purpose of this study, and manually placed in the field. According to Dutch legislation, no ethical permits were needed for this research, since we only used wild ungulate carcasses. All carcasses were monitored until depletion, meaning that there were no carcass parts visible anymore.

Carcasses were only included in the analysis if the whole decomposition process was monitored (i.e., until complete decomposition and without gaps in video monitoring). These videos were annotated—via the online platform Agouti (WUR and INBO 2025)—to determine (1) the scavenger species and the number of individuals; (2) their behaviour (Table 1); (3) if applicable, the consumed or collected body tissues (Table 2); and (4) the stage of decomposition at the time of scavenger visitation (Table 3) (Wenting et al. 2022). In the case that two or more types of species visited a carcass simultaneously, the video was annotated for each species separately. In addition, the dates of placement and carcass depletion were noted per carcass to calculate the time to depletion, as well as the time until first detection and first scavenging (Wenting et al. 2022).

**TABLE 1:** Definitions used to annotate the behaviour of facultative scavengers recorded by the camera traps (reproduced from Wenting et al. 2022).

Behaviour	Abbreviation	Definition
Passing	PAS	Move in front of the camera trap without moving body and/or head in the direction of the carcass.
Interest	INT	Body and/or head moves toward the carcass, or mouth/beak touches the carcass without any chewing/picking movements.
Eating	EAT	Mouth/beak touches the carcass, and removing carcass parts by chewing/picking movements.
Standing on carcass	STA	Touching the carcass with legs only; i.e., no other body parts other than the legs touch the carcass.
Intraspecific interaction	INTRA	Physical and non‐physical contact between individuals of the same scavenger species.
Interspecific interaction	INTER	Physical and non‐physical contact between individuals of a different scavenger species.
Collecting material	CM	Taking along carcass parts.

**TABLE 2:** Tissue types annotated of carrion consumption by facultative scavengers recorded by the camera traps (reproduced from Wenting et al. 2022).

Tissue types
Bones and hooves
Hairs
Nose, ears, eyes, anus, and skin on the armpits and abdominal region (soft tissues)
Skin on other parts of the body
Muscle
Organs
Insects and larvae that were present on the carcass, i.e., indirect carcass consumption

**TABLE 3:** Definitions used to annotate the stages of decomposition scavengers recorded by the camera traps (Feddern et al. 2019; reproduced from Wenting et al. 2022).

Stage of decomposition	Definition
Bloated stage	The carcass is fresh and/or abdominal bloating occurs due to anaerobic microbial activity, and the carcass has no or only minor injuries that do not expose any entrails
Active decay	Characterised by rapid mass and volume loss due to increased scavenger activity, and during which at least some entrails are exposed
Advanced decay	Characterised by a flat abdomen and only some parts of the skin and skeleton remains, possibly supplemented by some other tissue leftovers

### 2.3 Statistical Analyses

The statistical analyses were determined based on the results of the previous step (Figure S1). All statistical analyses were done in R version 4.5.1 (R core team 2025). In total, we used 11,183 observations of facultative scavengers visiting the carcasses (Wenting, Jansen, et al. 2024): 8158 in the Veluwe, 2230 in De Hamert Estate, and 795 in KempenBroek. We observed a total of 49 species (Table S2).

First, we determined the regional functional scavenger groups by following the steps described by Wenting et al. (2022), in order to explore functional similarity among species. In short, (1) we selected the species for further analyses by including only those showing eating behaviour with a minimum of 30 observations per region (Wenting et al. 2022); (2) calculated the percentage of observations per species for each behaviour (Table 1), consumed tissue (Table 2), and decomposition stage (Table 3); and noted (3) the average time to first detection and time to first consumption per scavenger species; (4) as well as the adult body mass (grams) per scavenger species as a proxy of their capacity to tear open carcass skin. We used these data to perform principal component analyses (PCAs) in order to group the species per area, focusing on previously described scavenging characteristics (Tables 1, 2, 3). We aimed to compare the results with predefined functional groups (Wenting et al. 2022) rather than defining new ones. The PCAs were performed using the FactoMineR (Husson et al. 2017), factoextra (Kassambara and Mundt 2020) and ggplot2 (Wickham 2016) packages, which allow integration of both categorical and continuous variables and provide biplots for interpretation of species‐trait relationships. All variables were centered and scaled prior to PCA using (Husson et al. 2017) to ensure comparability across measurement units. We compared the PCAs per region with the functional scavenger groups described by Wenting et al. (2022), i.e., Wild boar, (other) Mammals, Birds, and Occasional scavengers. Interpretation was based on the position of species in the biplots, with species located close together sharing similar functional traits.

Second, we examined temporal variation in functional roles of facultative scavengers focusing on the Veluwe. We selected the Veluwe region for this analysis because it contained the highest number of carcasses in subsequent years: nine in both 2020 and 2021, enabling us to detect fine‐scale changes. We conducted additional PCAs to examine how the monitoring period influenced the outcome for this region. We only included the Wild boar, (other) Mammals and Birds (i.e., Specialists) in the analyses based on the selection criterium of a minimum of 30 observations per species.

Last, to examine the effect of scavenger diversity on the speed of carrion decomposition, we plotted diversity as Shannon index against the time to carcass depletion with fitted linear regression lines for each of the three regions. Therefore, we used linear mixed models with carcass depletion time as the dependent variable and scavenger diversity index as the independent variable. We repeated this for consecutive monitoring years in the Veluwe region.

## 3 Results

### 3.1 Functional Scavenger Groups Per Region

The annotated data included information on scavenger species identity, behaviour, tissue consumption, and timing of carcass visitation (Wenting et al. 2022; Wenting, Jansen, et al. 2024; Tables S3–S5). These variables showed variation among species and regions, which formed the basis for the functional trait analyses presented in the PCAs.

**FIGURE 2:**
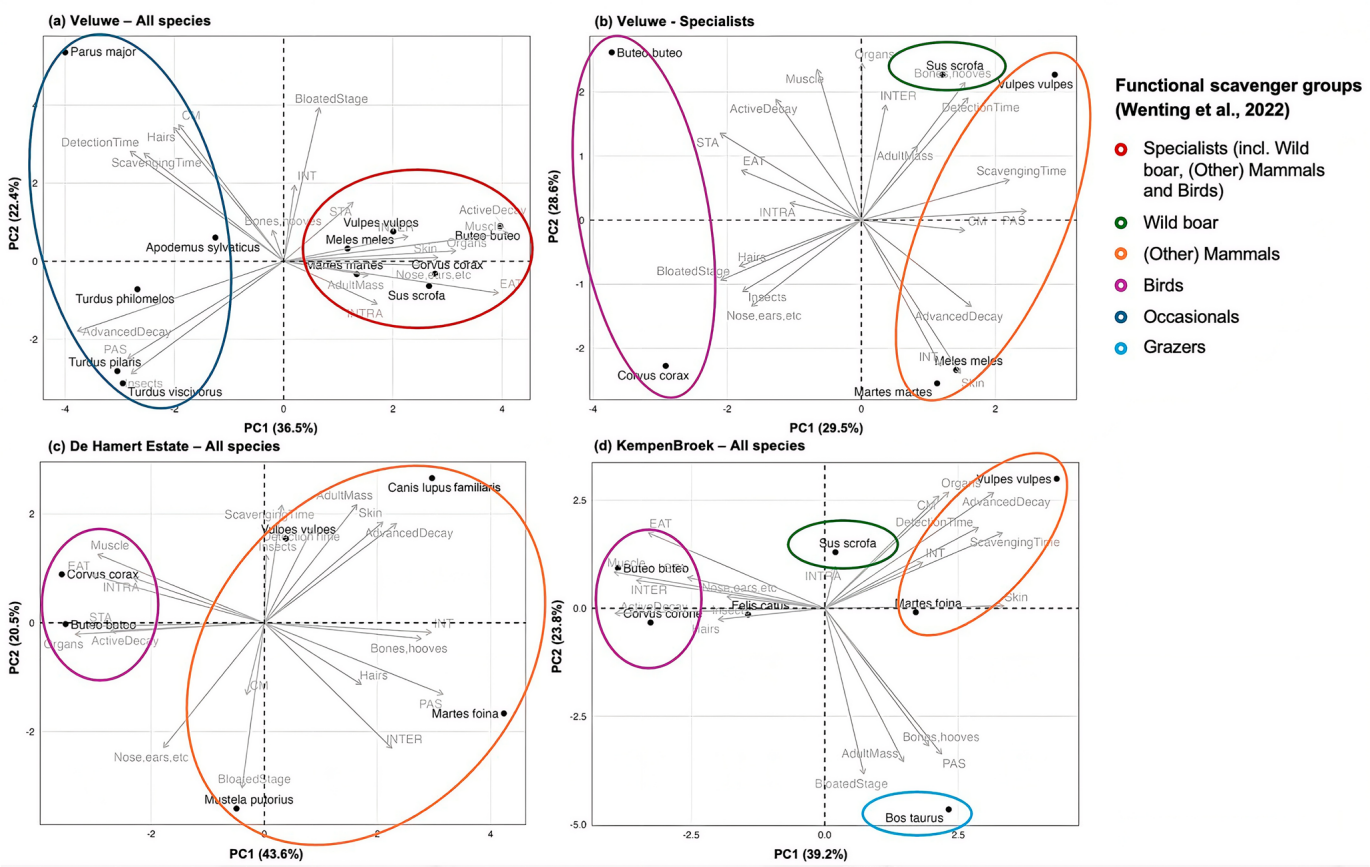
PCA biplots of (a) all species found in the Veluwe, (b) further analysis of the specialist species in the Veluwe (red circle in panel a), (c) all species found in De Hamert Estate, and (d) all species found in KempenBroek. The circles indicate the functional groups as defined by Wenting et al. (2022).

Based on the PCAs, we found that scavengers categorised as Occasionals (Wenting et al. 2022) only occurred at the Veluwe (Figure 2a) and not at the other two study regions. This included the great tit, wood mouse ( *Apodemus sylvaticus* ), song thrush ( *Turdus philomelos* ), fieldfare ( *Turdus pilaris* ), and mistle thrush ( *Turdus viscivorus* ). An additional PCA for specialists, i.e., excluding Occasionals (Wenting et al. 2022), in the Veluwe region showed that red fox and wild boar were located rather close to each other on the plot (Figure 2b).

Two functional scavenger groups were found in De Hamert Estate: Birds and (other) Mammals (Figure 2c). The (other) Mammals showed higher diversity compared to the other regions.

The KempenBroek showed a comparable functional diversity as the Veluwe, despite occasional scavengers being absent but grazers (domestic cattle) being present (Figure 2d).

### 3.2 Changes in Functional Scavenger Groups Over Time

**FIGURE 3:**
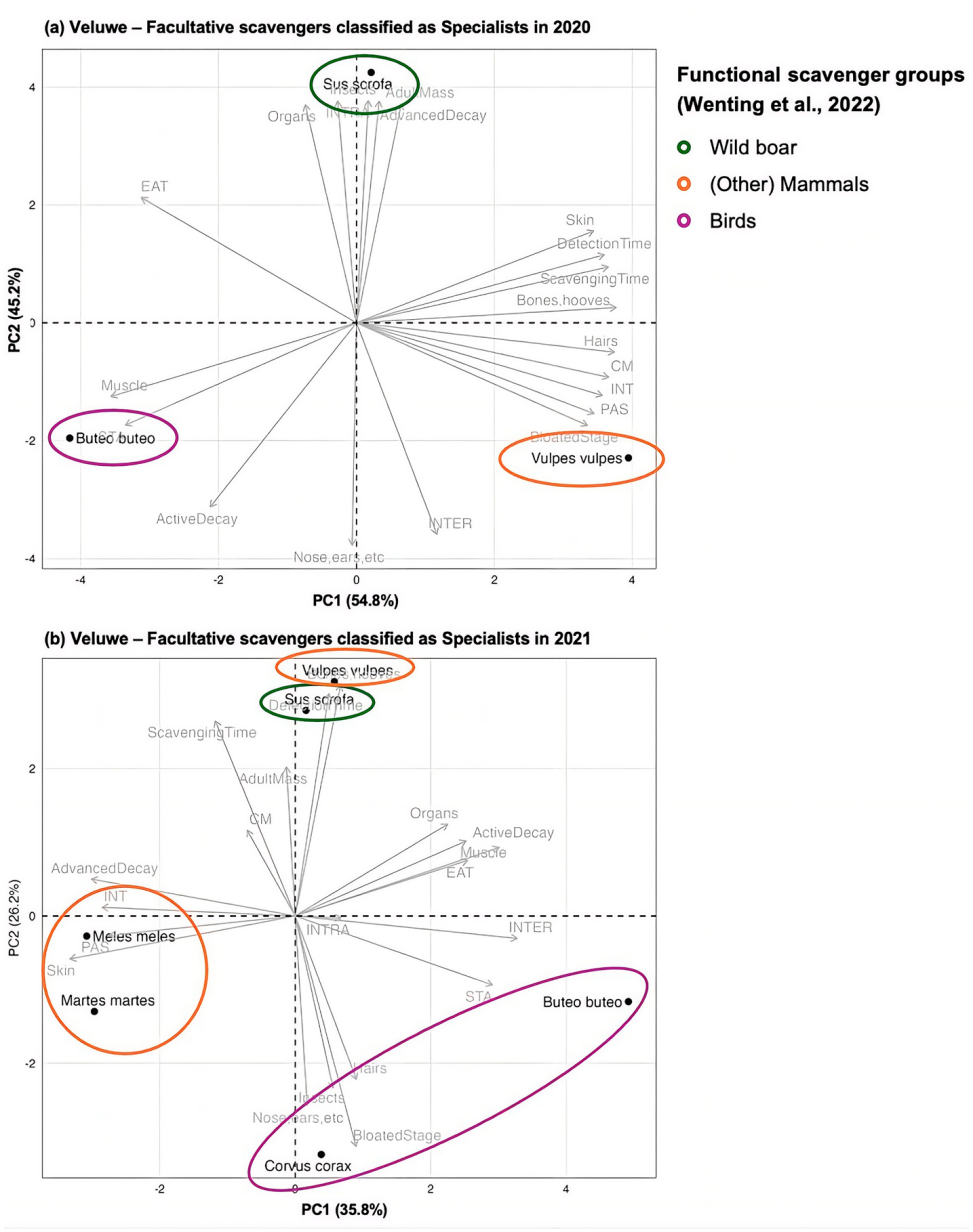
PCA biplots of the specialist scavenger species found on the Veluwe from the study period in 2020 (a) and 2021 (b). The circles indicate the functional groups as defined by Wenting et al. (2022).

Focussing on the Veluwe region, we conducted additional PCAs to examine how the monitoring period in subsequent years influenced the outcome for this region. This revealed that the red fox showed different scavenging behaviour in 2020 (Figure 3a)—more associated with interaction with other species and with earlier decomposition stages—than in 2021 (Figure 3b)—more associated with fast carcass detection and later decomposition stages, for example, feeding on bones and hooves.

### 3.3 Scavenger Diversity Versus Carcass Depletion Time

**FIGURE 4:**
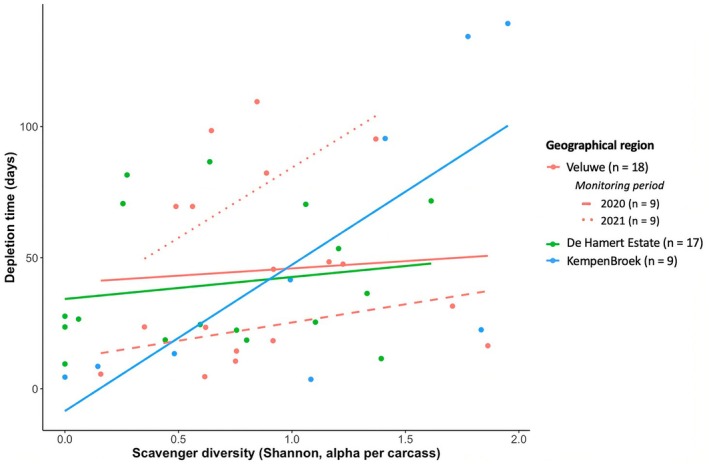
Scavenger diversity versus the time until carcass depletion per region for all scavenger species. For the Veluwe, additional trendlines (dotted and striped) are added to separate between monitoring periods.

Overall, higher scavenger diversity was positively correlated with longer decomposition times (LMM, df = 30.555, *F* = 17.255, *p* = 0.0002). We found the strongest trend for the KempenBroek region (Figure 4; LMM, df = 7, *F* = 7.984, *p* = 0.026). We observed a slightly positive but not significant trend for both De Hamert Estate (Figure 4; LMM, df = 15, *F* = 0.455, *p* = 0.510) and the Veluwe (Figure 4; LMM, df = 16, *F* = 0.083, *p* = 0.777). For the Veluwe, we distinguished between two monitoring years—2020 and 2021—and observed a stronger but not significant correlation between scavenger diversity and carcass depletion time in 2021 (Figure 4; LMM, df = 6, *F* = 1.824, *p* = 0.226) compared to 2020 (Figure 4; LMM, df = 5, *F* = 0.451, *p* = 0.532).

## 4 Discussion

This study aimed to determine the functionality of scavenger guilds on a regional and temporal scale. We used a dataset of Dutch scavenger communities (Wenting et al. 2022; Wenting, Jansen, et al. 2024) to evaluate scavengers' functional roles, scavenger diversity, and carrion depletion times. We found high consistency in functional traits represented in the regions (Figure 1), although Occasional scavengers, were only present at the Veluwe (Figure 2a,b). This seems in line with the overall higher species diversity in the Veluwe region compared to the other regions (e.g., Rutgers et al. 2019; Veen et al. 2024).

The functionality of facultative scavenger communities may temporally vary within a region, as we showed for the Veluwe region (Figure 3). Red fox and wild boar showed notably more overlap in functional scavenging behaviour in 2021 compared to 2020, particularly associated with carrion detection time and consumption of bony material. Red fox has been globally described as flexible and highly opportunistic, that can quickly adapt to locally and temporally changing circumstances (Díaz‐Ruiz et al. 2016; Morton et al. 2023). Our result may be the indirect consequence of changes in food availability between the years of monitoring (Young et al. 2015), although we do not have the data to prove this assumption. Similarly, feeding behaviour of wild boar is strongly related to oak and beech masts (e.g., Bisi et al. 2018; Erdtmann and Keuling 2020). Consequently, the temporal circumstances during the period of monitoring—e.g., lots of mast in 2020 but not in 2021—may have contributed to smaller differences in scavenging behaviour between red fox and wild boar, indicating that the functionality of scavenger communities can temporarily differ within regional scales.

We observed an overall increasing trend between scavenger diversity and depletion time (Figure 4), indicating that longer carrion persistence allows more species to utilise it (Wenting et al. 2022). The wide variety in carrion depletion times, particularly in the KempenBroek region, may imply that carrion decomposition has different local effects on, for instance, nutrient leakage into the soil beneath the carrion (Wenting et al. 2023). We noticed longer depletion times in the Veluwe region in 2021 compared to 2020, while the scavenger diversity was rather similar. This variability could reflect differences in local conditions and temporal variation, such as food availability or environmental conditions (Spencer et al. 2024; Grootaers et al. 2025; Monar‐Barragán et al. 2025). However, because monitoring periods differed among regions, regional and temporal effects are partly confounded, and some of the observed differences between regions may be confounded by temporal variation. Yet, our results emphasise the variability and unpredictability of carrion decomposition (e.g., Moleón et al. 2019; Wenting et al. 2022; Bartel et al. 2024), which highlights the relevance of examining functionality of scavenger communities across regions and over time.

Our results indicate that functionality of scavenger communities depends on regional and temporal scales. This underlines the importance of continuously or periodically monitoring the functionality of scavenger communities, given that carrion decomposition is a key ecological process that is paramount to other fundamental ecological processes, including nutrient recycling (e.g., Barton et al. 2013; Bartel et al. 2024; DeBruyn et al. 2025). Given the rapid change of vertebrate communities across European ecosystems (Wenting, Eikelboom, et al. 2024), for example due to the re‐establishment of large carnivores (e.g., Planillo et al. 2024), we propose that functionality of scavenger communities can serve as a key indicator of changing ecological processes. Given that our study system lacked large carnivores and obligate scavengers, this study can be considered as representative of similar European landscapes and serve as a baseline for evaluating changes when large carnivores and/or obligate scavengers are re‐establishing.

## 5 Conclusion

In conclusion, our study demonstrates that similar functional roles of facultative scavengers are represented in an ecosystem without obligate scavengers and large carnivores, although there are region‐specific differences due to species occurrence. Temporal differences may be influenced by changes in availability of other resources and species' flexibility to adapt to local and temporal circumstances, as suggested by our comparison of consecutive monitoring years in the Veluwe region. We found higher scavenger diversity with longer carrion persistence across all regions. Overall, these findings highlight the importance of continuous or periodic monitoring of the functionality of local scavenger communities, as an indication for altered ecological function due to changes in species composition and other ecological processes.

## Author Contributions

**Indy van der Giessen:** conceptualization (lead), formal analysis (lead), investigation (equal), methodology (equal), visualization (lead), writing – original draft (lead), writing – review and editing (equal). **Julia van Knippenberg:** conceptualization (equal), project administration (equal), supervision (lead), writing – review and editing (supporting). **Marijke van Kuijk:** conceptualization (supporting), supervision (supporting), writing – review and editing (supporting). **Elke Wenting:** conceptualization (equal), data curation (equal), investigation (supporting), methodology (lead), project administration (equal), resources (lead), validation (lead), writing – original draft (supporting), writing – review and editing (equal).

## Conflicts of Interest

The authors declare no conflicts of interest.

## Supporting information


**Table S1:** The number and types of carcasses used in each geographical region.
**Figure S1:** The workflow of statistical analyses and corresponding outcomes.
**Table S2:** Number of observations of each facultative scavenger species per geographical region.
**Table S3:** Percentage of observations per decomposition stage, behaviour, and tissue type, per selected species in the Veluwe region that we used in the PCA.
**Table S4:** Percentage of observations per decomposition stage, behaviour, and tissue type, per selected species in De Hamert Estate region that we used in the PCA.
**Table S5:** Percentage of observations per decomposition stage, behaviour, and tissue type, per selected species in the KempenBroek region that we used in the PCA.

## Data Availability

The data used in this study originated from Wenting, Jansen, et al. (2024) and can be accessed via https://doi.org/10.6084/m9.figshare.23634000. We used the carcasses monitored in three geographical regions: Veluwe (Planken Wambuis and Veluwezoom National Park); De Hamert Estate; and KempenBroek (Grenspark Kempen~Broek and Valkenhorst Estate).
