# Supplementary material for: Regional and Temporal Differences in the Functionality of Facultative Vertebrate Scavenger Communities
Source: Ecol Evol. 2026 Jun 3;16(6):e73625. doi: 10.1002/ece3.73625 (PMC13240032; doi:10.1002/ece3.73625)

**Supplementary Materials**

Table S1 The number and types of carcasses used in each geographical region.

|  | **Veluwe** | **De Hamert Estate** | **KempenBroek** |
| --- | --- | --- | --- |
| Wild boar | 8 | - | 1 |
| Roe deer | - | 15 | 7 |
| Fallow deer | 7 | - | - |
| Red deer | 3 | - | - |
| European badger | - | 1 | 1 |
| Sheep (Ovis orientalis) | - | 1 | - |
| **Total** | **18** | **17** | **9** |

Table S2 The counts of scavenger species observed in each geographical region.

| **Species**  Total nr of different species | **Veluwe**  n = 34 | **De Hamert Estate** n = 26 | **KempenBroek** n = 20 |
| --- | --- | --- | --- |
| Accipiter gentilis | 1 | 1 | - |
| Alopochen aegyptiaca | - | 2 | - |
| Anas platyrhynchos | - | 2 | 1 |
| Anser anser | - | 24 | 1 |
| Apodemus sylvaticus | 190 | - | - |
| Ardea cinerea | - | 13 | - |
| Bos taurus | 41 | - | 52 |
| Branta canadensis | - | 2 | - |
| Bufo bufo | - | 1 | - |
| Buteo buteo | 1104 | 198 | 92 |
| Canis lupus familiaris | 1 | 39 | 4 |
| Capreolus capreolus | 12 | 16 | 15 |
| Certhia brachydactyla | 1 | - | - |
| Cervus elaphus | 4 | - | - |
| Coccothraustes coccothraustes | 2 | - | - |
| Columba palumbus | 1 | 1 | 1 |
| Corvus corax | 2044 | 857 | - |
| Corvus corone | 16 | 10 | 170 |
| Cyanistes caeruleus | 1 | - | - |
| Erinaceus europaeus | - | - | 11 |
| Erithacus rubecula | 18 | - | - |
| Felis catus | - | 5 | 56 |
| Fringilla coelebs | 2 | - | - |
| Fringilla montifringilla | 1 | - | - |
| Garrulus glandarius | 5 | 2 | - |
| Lepus europaeus | - | - | 37 |
| Lullula arborea | 4 | - | - |
| Martes foina | - | 47 | 26 |
| Martes martes | 52 | - | - |
| Meles meles | 34 | 9 | - |
| Motacilla alba | 1 | 117 | - |
| Mustela putorius | - | - | 1 |
| Oenanthe oenanthe | 1 | - | - |
| Oryctolagus cuniculus | - | 5 | 2 |
| Ovis aries | - | 40 | - |
| Parus major | 33 | 2 | 13 |
| Passer domesticus | 2 | - | - |
| Phasianus colchicus | - | - | 1 |
| Pica pica | - | 5 | - |
| Sciurus vulgaris | 2 | 1 | 14 |
| Sitta europaea | 1 | - | - |
| Strix aluco | 1 | - | - |
| Sus scrofa | 3588 | - | 239 |
| Turdus merula | 2 | 12 | 2 |
| Turdus philomelos | 262 | 3 | - |
| Turdus pilaris | 30 | - | - |
| Turdus viscivorus | 36 | - | - |
| Vulpes vulpes | 664 | 816 | 57 |
| Zootoca vivipara | 1 | - | - |
| **Total** | 8158 | 2230 | 795 |

Table S3 Percentage of observations per decomposition stage, behaviour, and tissue type, per selected species for the Veluwe

|  | **Bloated stage** | **Active decay** | **Advanced decay** | **CM** | **EAT** | **INT** | **INTER** | **INTRA** | **PAS** | **STA** | **Bones** | **Hairs** | **Insects** | **Muscle** | **Soft** | **Organs** | **Skin** | **Detection time** | **Time till scavenging** | **Adult body mass** |
| --- | --- | --- | --- | --- | --- | --- | --- | --- | --- | --- | --- | --- | --- | --- | --- | --- | --- | --- | --- | --- |
| *A. sylvaticus* | 5.8 | 13.2 | 81.1 | 0.0 | 20.6 | 58.8 | 0.0 | 0.8 | 15.5 | 4.2 | 60.0 | 0.0 | 6.7 | 26.7 | 0.0 | 0.0 | 6.7 | 21125.985 | 41562.632 | 21.9 |
| *B. buteo* | 15.3 | 64.9 | 19.7 | 0.1 | 58.6 | 19.9 | 0.6 | 1.1 | 1.4 | 18.4 | 2.2 | 1.7 | 1.0 | 71.9 | 6.4 | 3.1 | 13.7 | 3585.696 | 5260.848 | 875 |
| *C. corax* | 14.3 | 37.8 | 47.9 | 0.9 | 58.8 | 31.8 | 0.3 | 5.6 | 1.6 | 1.1 | 0.8 | 9.0 | 10.7 | 14.0 | 22.5 | 2.6 | 40.4 | 5146.323 | 5146.323 | 1200 |
| *M. martes* | 11.5 | 34.6 | 53.8 | 2.9 | 42.6 | 35.3 | 0.0 | 0.0 | 19.1 | 0.0 | 0.0 | 0.0 | 0.0 | 29.4 | 11.8 | 0.0 | 58.8 | 3883.968 | 5380.992 | 1675 |
| *M. meles* | 11.8 | 41.2 | 47.1 | 0.0 | 43.5 | 45.7 | 0.0 | 0.0 | 10.9 | 0.0 | 0.0 | 0.0 | 0.0 | 30.0 | 0.0 | 0.0 | 70.0 | 1452.43 | 4318.32 | 1390 |
| *P. major* | 27.3 | 3.0 | 69.7 | 35.7 | 7.1 | 33.3 | 0.0 | 0.0 | 19.0 | 4.8 | 5.9 | 94.1 | 0.0 | 0.0 | 0.0 | 0.0 | 0.0 | 66135.528 | 71979.024 | 19.25 |
| *S. scrofa* | 5.3 | 37.3 | 57.4 | 0.4 | 65.8 | 20.5 | 0.1 | 5.3 | 7.9 | 0.0 | 12.3 | 1.2 | 0.0 | 47.0 | 2.4 | 4.4 | 32.7 | 16834.554 | 26722.1472 | 84471 |
| *T. philomelos* | 5.0 | 0.4 | 94.7 | 1.9 | 20.4 | 49.8 | 0.0 | 1.3 | 24.5 | 2.2 | 0.0 | 11.5 | 88.5 | 0.0 | 0.0 | 0.0 | 0.0 | 24643.968 | 34886.448 | 67.75 |
| *T. pilaris* | 0.0 | 0.0 | 100.0 | 0.0 | 30.2 | 0.0 | 0.0 | 0.0 | 69.8 | 0.0 | 0.0 | 0.0 | 100.0 | 0.0 | 0.0 | 0.0 | 0.0 | 14348 | 17311 | 106 |
| *T. viscivorus* | 0.0 | 0.0 | 100.0 | 0.0 | 16.7 | 7.1 | 0.0 | 2.4 | 73.8 | 0.0 | 0.0 | 0.0 | 100.0 | 0.0 | 0.0 | 0.0 | 0.0 | 14066.064 | 14066.064 | 117.5 |
| *V. vulpes* | 9.8 | 49.5 | 40.7 | 3.3 | 36.2 | 31.2 | 1.3 | 0.4 | 27.6 | 0.0 | 15.4 | 0.9 | 0.0 | 40.4 | 4.0 | 4.7 | 34.5 | 19695.24 | 33238.92 | 4820 |

Table S4 Percentage of observations per decomposition stage, behaviour, and tissue type, per selected species for the Hamert Estate

|  | **Bloated stage** | **Active decay** | **Advanced decay** | **CM** | **EAT** | **INT** | **INTER** | **INTRA** | **PAS** | **STA** | **Bones** | **Hairs** | **Insects** | **Muscle** | **Soft** | **Organs** | **Skin** | **Detection time** | **Time till scavenging** | **Adult body mass** |
| --- | --- | --- | --- | --- | --- | --- | --- | --- | --- | --- | --- | --- | --- | --- | --- | --- | --- | --- | --- | --- |
| *B. buteo* | 24.2 | 6.6 | 69.2 | 0.0 | 67.6 | 20.2 | 0.4 | 6.1 | 3.6 | 2.0 | 2.3 | 3.5 | 0.0 | 30.4 | 36.3 | 22.2 | 5.3 | 15129.014 | 12627.504 | 875 |
| *C. lupus familiaris* | 2.6 | 92.3 | 5.1 | 0.0 | 51.7 | 28.3 | 1.7 | 1.7 | 16.7 | 0.0 | 14.3 | 0.0 | 0.0 | 4.8 | 0.0 | 4.8 | 76.2 | 15357.024 | 12627.504 | 35000 |
| *C. corax* | 1.6 | 52.2 | 46.2 | 4.6 | 68.9 | 3.2 | 0.5 | 16.1 | 4.3 | 2.4 | 2.4 | 4.7 | 0.3 | 44.6 | 9.1 | 17.7 | 21.3 | 2331.984 | 7659.648 | 1200 |
| *M. foina* | 17.0 | 57.4 | 25.5 | 3.1 | 21.5 | 44.6 | 3.1 | 1.5 | 26.2 | 0.0 | 50.0 | 25.0 | 0.0 | 0.0 | 0.0 | 0.0 | 25.0 | 3452.12 | 3452.12 | 1675 |
| *M. putorius* | 43.6 | 35.0 | 21.4 | 5.6 | 57.6 | 16.0 | 2.8 | 0.0 | 17.4 | 0.7 | 3.8 | 0.0 | 0.0 | 3.8 | 52.8 | 15.1 | 24.5 | 2530.08 | 2530.08 | 975.55 |
| *V. vulpes* | 8.1 | 58.7 | 33.2 | 5.7 | 54.3 | 27.8 | 0.2 | 0.3 | 11.7 | 0.0 | 24.7 | 1.6 | 2.8 | 21.5 | 7.1 | 11.6 | 30.6 | 8365.286 | 18084.456 | 4820 |

Table S5 Percentage of observations per decomposition stage, behaviour, and tissue type, per selected species for KempenBroek

|  | **Bloated stage** | **Active decay** | **Advanced decay** | **CM** | **EAT** | **INT** | **INTER** | **INTRA** | **PAS** | **STA** | **Bones** | **Hairs** | **Insects** | **Muscle** | **Soft** | **Organs** | **Skin** | **Detection time** | **Time till scavenging** | **Adult body mass** |
| --- | --- | --- | --- | --- | --- | --- | --- | --- | --- | --- | --- | --- | --- | --- | --- | --- | --- | --- | --- | --- |
| B. taurus | 59.6 | 7.7 | 32.7 | 0.0 | 5.7 | 24.3 | 0.0 | 1.4 | 68.6 | 0.0 | 50.0 | 0.0 | 0.0 | 0.0 | 0.0 | 0.0 | 50.0 | 66582 | 133489.003 | 613000 |
| B. buteo | 0.0 | 2.2 | 97.8 | 0.8 | 51.2 | 22.0 | 4.9 | 0.8 | 12.2 | 8.1 | 0.0 | 0.0 | 0.0 | 95.2 | 0.0 | 0.0 | 4.8 | 18731.328 | 5557.968 | 875 |
| C. corone | 18.8 | 8.2 | 72.9 | 1.8 | 38.6 | 36.1 | 2.2 | 0.4 | 20.2 | 0.7 | 2.6 | 6.4 | 7.7 | 60.3 | 17.9 | 0.0 | 5.1 | 2817.648 | 3872.304 | 375 |
| F. catus | 14.3 | 19.6 | 66.1 | 0.0 | 49.4 | 29.6 | 1.2 | 0.0 | 19.8 | 0.0 | 0.0 | 0.0 | 0.0 | 54.8 | 0.0 | 0.0 | 45.2 | 5672.52 | 921.024 | 2885 |
| M. foina | 3.8 | 38.5 | 57.7 | 0.0 | 9.8 | 51.2 | 0.0 | 0.0 | 39.0 | 0.0 | 0.0 | 0.0 | 0.0 | 0.0 | 0.0 | 0.0 | 100.0 | 53873.43 | 53873.43 | 1675 |
| S. scrofa | 13.8 | 38.1 | 48.1 | 1.0 | 44.2 | 16.5 | 0.2 | 30.1 | 7.9 | 0.0 | 7.0 | 0.0 | 0.0 | 34.4 | 9.6 | 16.6 | 32.5 | 12806.664 | 35175.84 | 84471 |
| V. vulpes | 0.0 | 91.2 | 8.8 | 8.8 | 18.7 | 46.2 | 0.0 | 1.1 | 25.3 | 0.0 | 10.0 | 0.0 | 0.0 | 0.0 | 0.0 | 20.0 | 70.0 | 39587.365 | 45528.542 | 4820 |

Figure S1 The workflow of statistical analyses and corresponding outcomes.


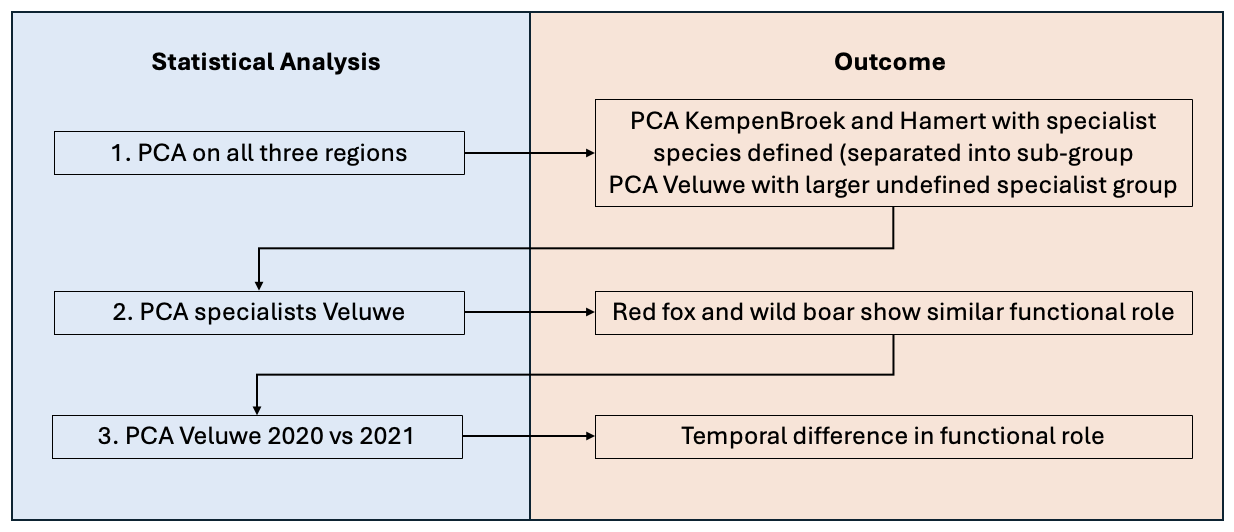

Supplement: Supplementary file 1 — Table S1: The number and types of carcasses used in each geographical region. Figure S1: The workflow of statistical analyses and corresponding outcomes. Table S2: Number of observations of each facultative scavenger species per geographical region. Table S3: Percentage of observations per decomposition stage, behaviour, and tissue type, per selected species in the Veluwe region that we used in the PCA. Table S4: Percentage of observations per decomposition stage, behaviour, and tissue type, per selected species in De Hamert Estate region that we used in the PCA. Table S5: Percentage of observations per decomposition stage, behaviour, and tissue type, per selected species in the KempenBroek region that we used in the PCA. [file ECE3-16-e73625-s001.docx]
